# Supplementary material for: Independent Avian Epigenetic Clocks for Ageing and Development
Source: Mol Ecol Resour. 2025 Jun 3;25(7):e14128. doi: 10.1111/1755-0998.14128 (PMC12415945; doi:10.1111/1755-0998.14128)

**Supplemental Information for:**

**Independent avian epigenetic clocks for aging and development**

Ayke Haller^1,2^, Judith Risse^1,3^, Bernice Sepers^1,2,4^ & Kees van Oers^1,2^ ^*^

1. Department of Animal Ecology, Netherlands Institute of Ecology (NIOO-KNAW), Wageningen, the Netherlands
2. Behavioural Ecology Group, Wageningen University & Research (WUR), Wageningen, the Netherlands
3. Bioinformatics Group, Wageningen University & Research (WUR), Wageningen, the Netherlands
4. Department of Evolutionary Population Genetics, Bielefeld University, Bielefeld, Germany

Figure S1. Histogram of the number of unmerged CpGs from 134 post-fledging samples. The red line indicates the cut-off point of 12 samples that were removed from the analysis.


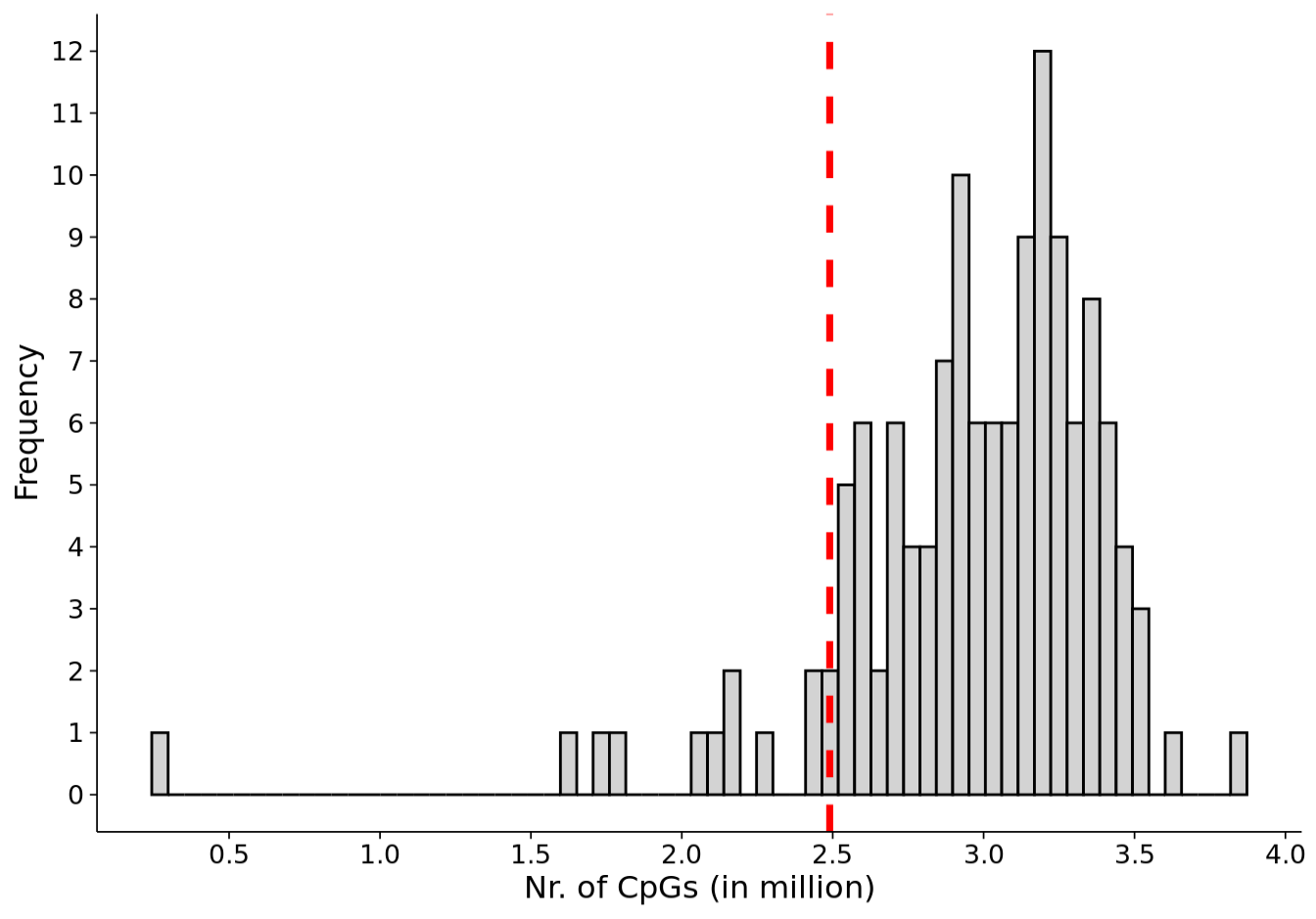


Table S1. The number of CpGs of the post-fledging datafiles and the number of remaining CpGs after each filtering step. Coverage filter, high percentile of 99.9% filter, filter for mean methylation more than 5% and less than 95%, and filter for standard deviation of more than 0.05, were only applied to the CpGs that were covered throughout the remaining 122 samples.

|  |  | Average | *N* samples |
| --- | --- | --- | --- |
| Total CpGs |  | 15,372,018 | 134 |
| Empty CpGs removed |  | 2,963,751 | 134 |
| Merged CpGs |  | 2,552,100 | 122 |
| Selected CpGs only in all samples |  |  |  |
| CpGs (10x min. coverage & high percentile filter of 99.9%) |  | 35,126 | 122 |
| Mean & SD filter |  | 8,398 | 122 |


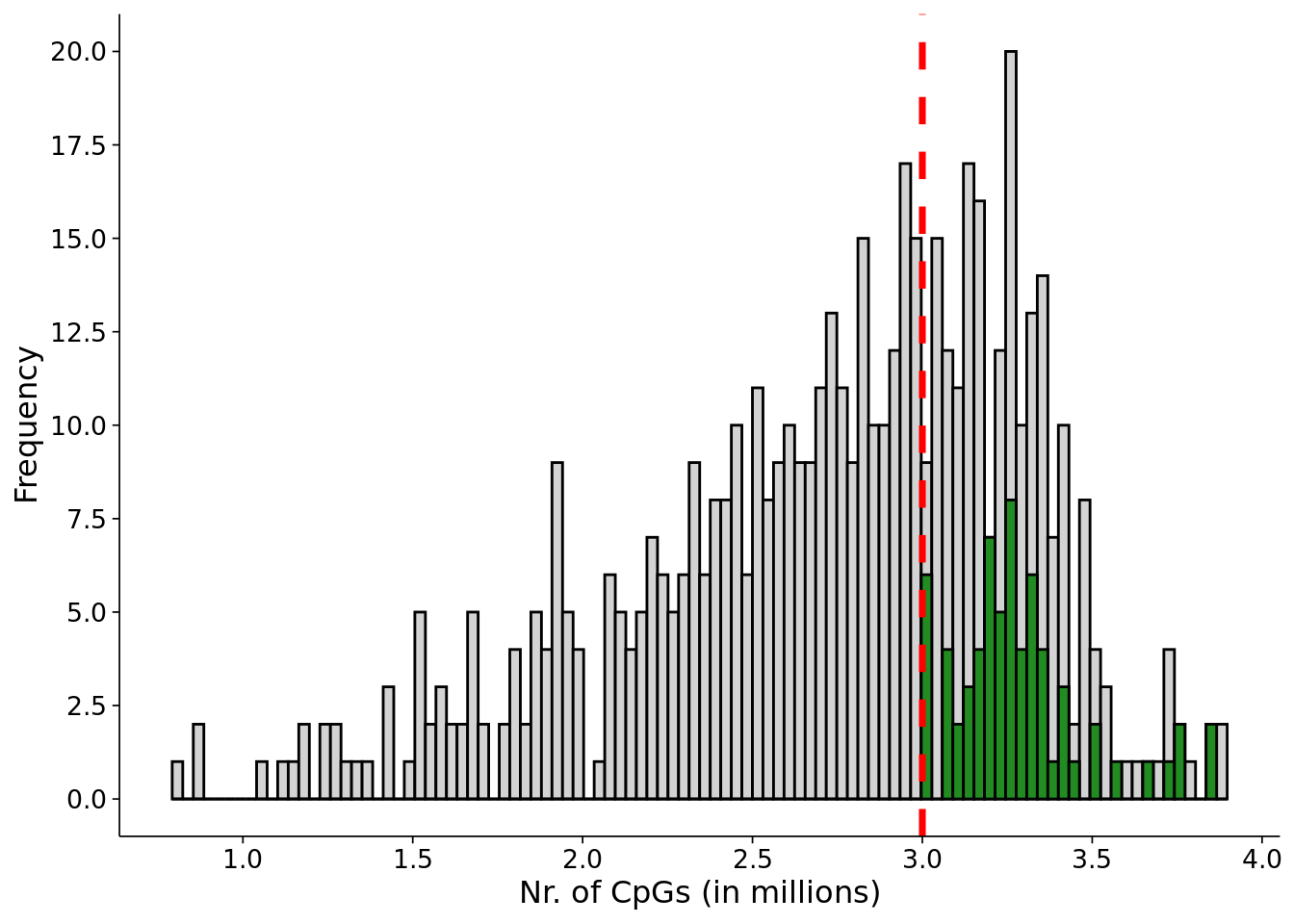


Figure S2. Histogram of the number of unmerged CpGs from 618 pre-fledging samples. The red line indicates the cut-off point of 346 samples that were excluded from the analysis. Green bars indicate the 67 samples that were selected to construct the final development clock model.

Table S2. The number of CpGs of pre-fledging datafiles and the number of remaining CpGs after removing empty CpGs with 0 Cs and Ts, and the number of CpGs that were present in at least one sample after filtering for >10 coverage and a coverage over high percentile of 99.9%.

|  | Average | *N* samples |
| --- | --- | --- |
| Total CpGs | 15,372,018 | 618 |
| Empty s removed | 2,784,984 | 618 |
| Selected samples > 3 million CpGs |  |  |
| Merged & Filtered CpGs (10x min. coverage & high percentile filter of 99.9%) | 2,055,751 | 272 |


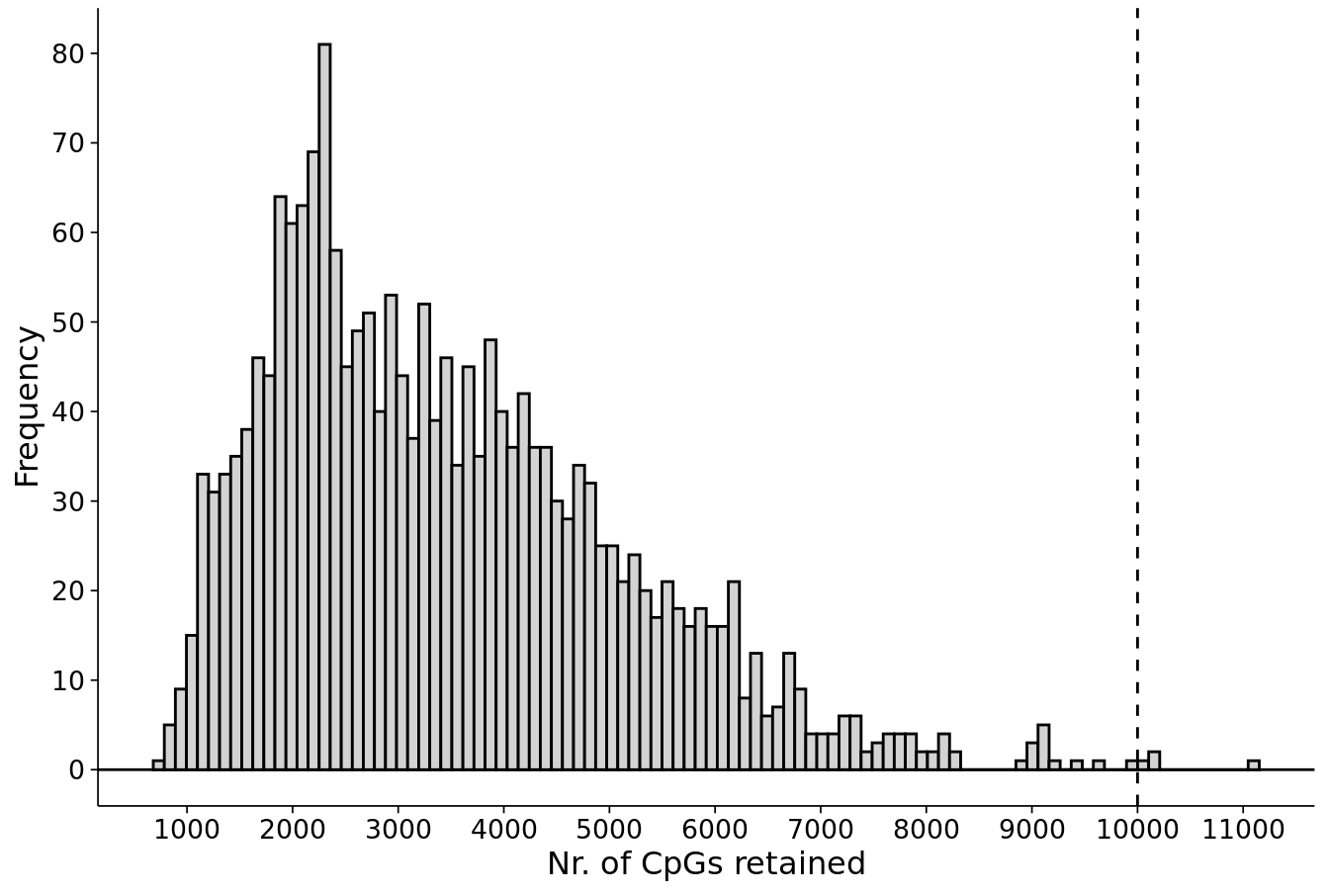


Figure S3. Histogram of numbers of CpGs that were retained in 500 randomised subsets of ~25% from 272 pre-fledglings. Four subsets were found that contained > 10,000 CpGs, indicated by the dashed lined.

Table S3. The number CpGs in the selected subset of pre-fledging individuals that were used to construct the final development epigenetic clock model.

|  | Number | *N* samples |
| --- | --- | --- |
| Total CpGs | 11,116 | 67 |
| Filtered CpGs (10x min. coverage & high percentile filter of 99.9%) | 3,184 | 67 |

Table S4. Number of pre-fledging samples per experiment that were used to construct the development epigenetic clock model.

|  | *N* | Age range in days | Males | Females | Unknown |
| --- | --- | --- | --- | --- | --- |
| All nestling samples | 67 | 6 - 15 | 26 | 25 | 16 |
| Per Experiment |  |  |  |  |  |
| Brood size manipulation | 34 | 14 – 15 | 20 | 14 |  |
| Testosterone manipulation | 17 | 6 – 7 | 6 | 11 |  |
| Food deprivation | 16 | 6 - 7 |  |  | 16 |


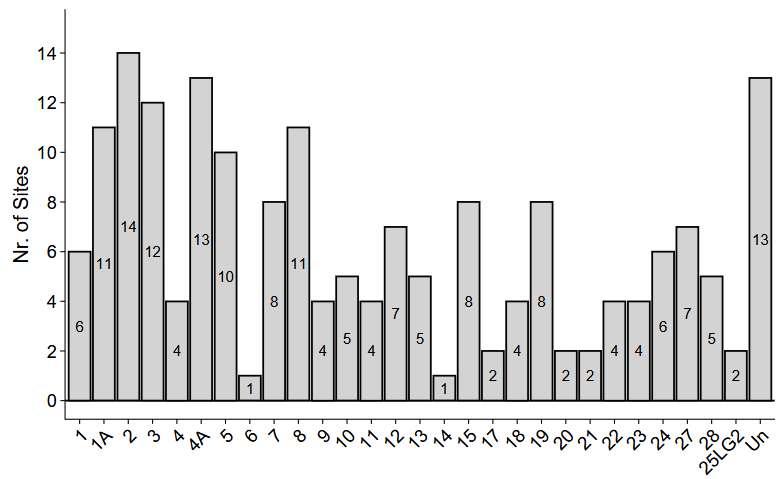


Figure S4. Histogram of the numbers of CpGs on each chromosome that were retained in the final aging clock model.


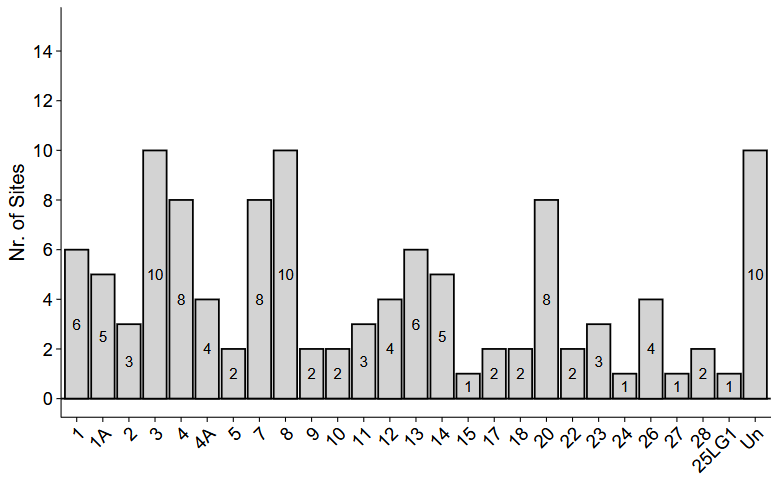


Figure S5. Histogram of the numbers of CpGs on each chromosome that were retained in the final development clock model.

Table S5. Total number of CpGs on each chromosome used to train the aging and development clock, and the number of selected CpGs in the final clock model. The *p*-values were computed using a two-sided Fisher’s Exact test for each chromosome separately, to test whether the selected CpGs were differently represented between the chromosomes.

|  | Aging | | | Development | | |
| --- | --- | --- | --- | --- | --- | --- |
| Chromosome | Total | Selected | *p*-value | Total | Selected | *p*-value |
| 1 | 397 | 6 | 0.480921 | 191 | 6 | 0.843572 |
| 1A | 330 | 11 | 0.180917 | 147 | 5 | 1 |
| 2 | 525 | 14 | 0.446032 | **269** | **3** | **0.016213** |
| 3 | 617 | 12 | 0.775848 | 228 | 10 | 0.586104 |
| 4 | 343 | 4 | 0.255754 | 139 | 8 | 0.24993 |
| 4A | **222** | **13** | **0.001815** | 92 | 4 | 0.777178 |
| 5 | 400 | 10 | 0.72672 | 154 | 2 | 0.124689 |
| 6 | **333** | **1** | **0.01098** | **112** | **0** | **0.033573** |
| 7 | 262 | 8 | 0.390198 | 141 | 8 | 0.253696 |
| 8 | **219** | **11** | **0.017548** | **103** | **10** | **0.006732** |
| 9 | 278 | 4 | 0.531137 | 92 | 2 | 0.775017 |
| 10 | 207 | 5 | 0.80938 | 84 | 2 | 0.769355 |
| 11 | 255 | 4 | 0.664064 | 111 | 3 | 0.797912 |
| 12 | 185 | 7 | 0.200317 | 70 | 4 | 0.340687 |
| 13 | 258 | 5 | 1 | 129 | 6 | 0.632779 |
| 14 | **261** | **1** | **0.047912** | 80 | 5 | 0.232765 |
| 15 | 415 | 8 | 0.863771 | 100 | 1 | 0.268324 |
| 17 | 259 | 2 | 0.13112 | 67 | 2 | 1 |
| 18 | 220 | 4 | 1 | 46 | 2 | 0.691557 |
| 19 | 273 | 8 | 0.402528 | 29 | 0 | 0.624437 |
| 20 | 289 | 2 | 0.097142 | **92** | **8** | **0.026129** |
| 21 | 108 | 2 | 1 | 12 | 0 | 1 |
| 22 | 110 | 4 | 0.316613 | 34 | 2 | 0.375452 |
| LGE22 | 31 | 0 | 1 | 1 | 0 | 1 |
| 23 | 223 | 4 | 1 | 138 | 3 | 0.486335 |
| 24 | 200 | 6 | 0.461135 | 68 | 1 | 0.517339 |
| 25LG1 | 34 | 0 | 1 | 11 | 1 | 0.357298 |
| 25LG2 | 66 | 2 | 0.660627 | 20 | 0 | 1 |
| 26 | **212** | **0** | **0.015384** | 54 | 4 | 0.155047 |
| 27 | **113** | **7** | **0.015642** | 68 | 1 | 0.517339 |
| 28 | 205 | 5 | 0.808234 | 65 | 2 | 1 |
| Z | 78 | 0 | 0.419308 | 33 | 0 | 0.631162 |
| Un | 470 | 13 | 0.419505 | 204 | 10 | 0.344132 |


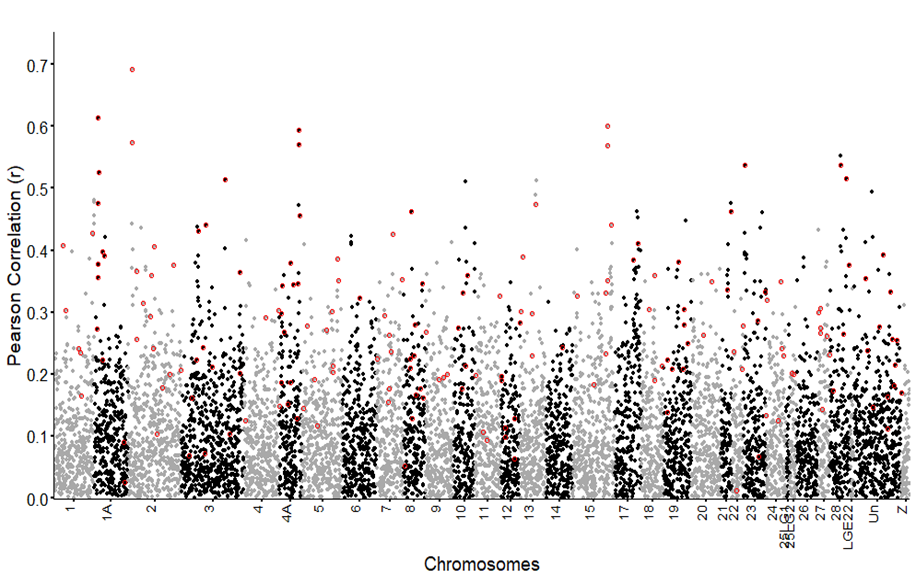


Figure S6. Manhattan plot of the pearson correlation (*r*) of each individual CpG site used to train the aging clock model. Each dot (N = 8398) represents a CpG site on their respective position on the chromosome. Red circles indicate the CpG sites that were retained in the final aging clock model (183). Overlapping sites represent chromosome 25LG1 and 25LG2, respectively.

Figure S7. Manhattan plot of the pearson correlation (*r*) of each individual CpG site used to train the development clock model. Each dot (N = 3184) represents a CpG site on their respective position on the chromosome. Red circles indicate the CpG sites that were retained in the final development clock model (N = 115). Overlapping sites represent chromosome 25LG1 and 25LG2, respectively.


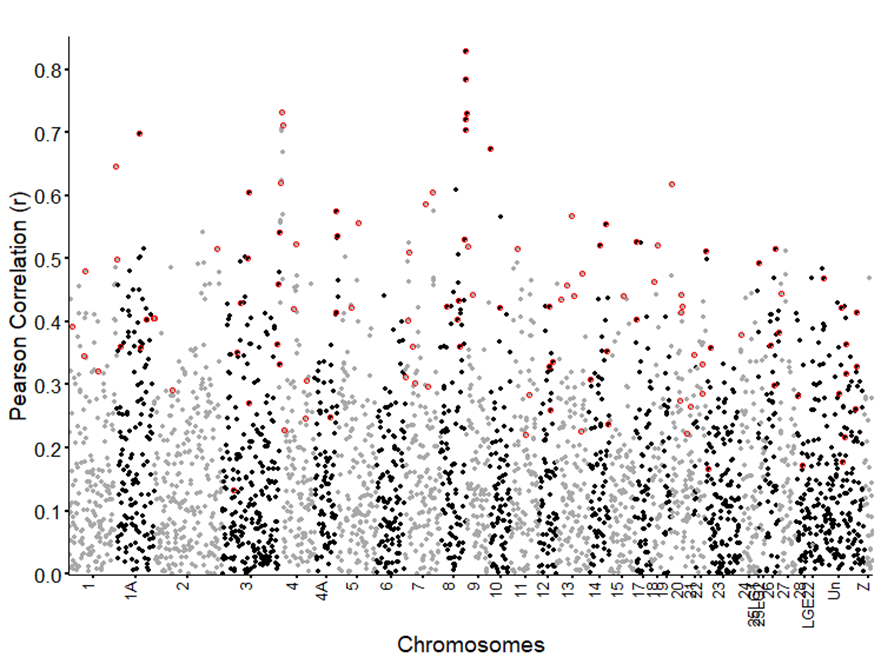

Supplement: Supplementary file 1 — Data S1. [file MEN-25-e14128-s002.docx]
